# Genome-wide characterisation of leaf rust resistance loci in an Eastern European barley collection

**DOI:** 10.1007/s00122-026-05405-4

**Published:** 2026-10-17

**Authors:** Shiva Azizinia, Md Arifuzzaman, Eden Tongson, Karanjeet Sandhu, Gabriel Keeble-Gagnère, Ghazanfar Abbas Khan, Kerrie Forrest, Matthew Hayden, Dragan Perovic, Davinder Singh, Peter M. Dracatos

**Affiliations:** 1 Agriculture Victoria, AgriBio, Centre for AgriBioscience, 5 Ring Rd, Bundoora, VIC 3083 Australia; 2 Department of Genetics and Plant Breeding, Hajee Mohammad Danesh Science and Technology University, Dinajpur, 5200 Bangladesh; 3 La Trobe Institute for Sustainable Agriculture and Food, Department of Ecological, Plant and Animal Sciences, La Trobe University, Bundoora, 3083 Australia; 4 Faculty of Science, Plant Breeding Institute, The University of Sydney, Cobbitty, NSW 2570 Australia; 5 School of Applied Systems Biology, 5 Ring Rd, La Trobe University, Bundoora, 3083 Australia; 6 School of Life and Environmental Sciences & Centre for Sustainable Bioproducts, Deakin University, Waurn Ponds, Victoria Australia; 7 Federal Research Centre for Cultivated Plants, Institute for Resistance Research and Stress Tolerance, Julius Kühn-Institut (JKI), Kleinmachnow, Germany

## Abstract

***Key message*:**

**GWAS and inheritance testing uncovered known and potentially new genetic sources of leaf rust resistance in barley, mapping the dominant locus** ***RphCaspian*** **(*RphCas*) to chromosome 4HL in close physical proximity to** ***Rph21.*** **This study has unravelled the genetic architecture of leaf rust in barley and also provides closely linked SNP markers on the 40k Infinium wheat barley array suitable for marker assisted selection.**

**Abstract:**

Barley leaf rust (BLR) is a damaging foliar disease affecting grain yield and quality. Genetic resistance offers a sustainable solution, but often succumbs to breakdown in the absence of stewardship. Sourcing and incorporating novel diverse alleles in breeding programs is challenging yet enhanced by mining unexplored gene pools available in gene banks. In this study, we investigated 318 barley accessions of Eastern European origin for BLR resistance using detailed greenhouse-based host–pathogen interaction assays at the seedling stage. The panel was genotyped using two marker platforms, targeted genotyping-by-sequencing and Infinium™ Wheat Barley 40 K BeadChip Array. Following marker curation, 35,059 SNPs were used for genome-wide association studies (GWAS) using GAPIT/FarmCPU, with associated regions further supported using a BayesR approach. GWAS identified significant peaks corresponding to known resistance loci, including *Rph2*,* Rph3* and *Rph12*, as well as potentially uncharacterised BLR resistance QTL on chromosomes 1HL, 2HL, 3HS and 6HS. Several biparental populations were also developed by crossing three diverse EEB donors carrying broadly effective BLR resistance including: Caspian, Anska and Athenais, with the leaf rust-susceptible barley cultivar Gus. Genetic analysis at the F_3_ generation revealed that resistance Athenais was consistent with two-gene inheritance, whereas resistance in Caspian was controlled by a single dominant gene (*RphCas*) and the inheritance of resistance in Anska was inconclusive. Bulked segregant RNA sequencing and conventional bulked segregant analysis (BSA) mapped the Caspian resistance locus to chromosome 4HL. An allelism test using an F_3_ population derived from Riccardo, carrying *Rph21*, and Caspian, tested with a *Puccinia hordei* pathotype (5457P+) avirulent to both resistance sources, susggested that the *RphCas* locus was distinct from *Rph21* however their close physical proximity and haplotypic similarity requires further investigation for a formal gene designation for *RphCas*.

**Supplementary Information:**

The online version contains supplementary material available at https://doi.org/10.1007/s00122-026-05405-4.

## Introduction

Barley leaf rust (BLR), caused by the fungus *Puccinia hordei* Otth, is a significant disease affecting barley crops worldwide. BLR is a major threat to barley production, requiring constant monitoring, effective management, and the development of resistant varieties to ensure food security and economic sustainability (Park et al. 2015). Severe infections can reduce barley yields by up to 40%, especially in susceptible varieties. The disease affects grain quality, lowering its value for food, feed, and malting (Cotterill et al. 1992; Murray and Brennan 2010). Rust pathogens have the ability to produce large numbers of urediniospores during the growing season that spread quickly under favourable conditions (warm, humid weather) and can evolve rapidly, overcoming previously effective resistance genes, making ongoing breeding efforts essential (Park 2003; Park et al. 2015).

Genetic resistance remains the most sustainable, cost-effective and environmentally sound strategy for managing BLR, but its long-term effectiveness depends on the identification and strategic deployment of diverse resistance alleles (Park et al. 2015; Dracatos et al. 2023; Arifuzzaman et al. 2023). Host resistance, particularly through the deployment of ***Rph*** genes (**R**esistance to ***P***. ***h****ordei*), offers durable protection, especially when used in combination (Singh et al. 2015; Rothwell et al. 2020; Chen et al. 2023; Dracatos et al. 2023). Two major forms of resistance are recognised: all-stage resistance (ASR) or seedling resistance, governed by *Rph* genes that provide strong, race-specific protection throughout the plant’s lifecycle and partial resistance that is termed adult plant resistance (APR) in the field, typically conferred by quantitative trait loci offering non-complete, generally non-race-specific resistance expressed later in development. To date, 28 *Rph* genes have been mapped and formally catalogued: 25 confer ASR (*Rph1–Rph19*, *Rph21*, *Rph22* and *Rph25–28*), whereas *Rph20*, *Rph23*, *Rph24* confer APR (Hickey et al. 2011; Ziems et al. 2017; Kavanagh et al. 2017; Rothwell et al. 2020; Mehnaz et al. 2021a), with additional QTL identified for partial resistance at the seedling stage (Qi et al. 1998; Niks et al. 2015) or adult plant stage (Singh et al. 2017).

Several studies have postulated the presence of particular known *Rph* genes through detailed multi-pathotype testing of core collections from various geographic origins including Africa, China and Central Asia either via the Australian Grains Genebank (AGG) or the CIMMYT Australia ICARDA Germplasm Evaluation (CAIGE) program (Derevnina et al. 2013; Elmansour et al. 2017; Mehnaz et al. 2021b; Singh et al. 2025). This is performed by generating infection type response data for each test genotype by testing with an array of 8–10 *P. hordei* pathotypes covering a broad spectrum of virulence/avirulence. Whereas broadly effective novel resistance allele identified at lower frequencies (usually a single or a few genotypes) potentially represent new source of resistance and subsequently hybridised (Resistant × Susceptible) to create mapping populations for inheritance studies, genetic mapping and allelism studies to rule out known *Rph* loci. Few studies however used a quantitative genetics approach to determine the presence of known and unknown genes in diversity collections.

Genebanks provide an essential pool of genetic diversity that can be used to source disease resistance alleles with the aim to enhance BLR resistance, ensuring sustainable and secure crop production (Milner et al. 2019). Breeding for disease resistance or other agronomically important traits in the western world is often influenced by human movement over time and collaboration. For example, due to political isolation, fewer alleles are likely to be sourced from eastern European origin (Arifuzzaman et al. 2023). Various population or genebank genomic approaches are currently used to enhance GWAS accuracy. Genome Association and Prediction Integrated Tool (GAPIT) has been designed for GWAS to identify genetic markers linked to important traits (like disease resistance, yield, etc.) in crops and other organisms. It has expanded functionality to improve statistical power, incorporating multi-locus GWAS methods such as Fixed and Random Model Circulating Probability Unification (FarmCPU). FarmCPU combines fixed and random effect models to control for both: population structure and multiple associated markers to avoid false positives and increase detection power to detect true associations. This model is distinct from other GWAS approaches as it uses an iterative process where significant markers are added as covariates (fixed effects), while controlling background variation through random effects (Kusmec and Schnable 2018).

In this study we report on the population genomic analysis of the Eastern European Barley (EEB) collection first assembled by Arifuzzaman et al. (2023). We genotyped 318 diverse EEB accessions using two genotypic platforms including both the Infinium™ Wheat Barley 40 K BeadChip (Keeble-Gagnère et al. 2021) and a targeted GBS platform and performed detailed quantitative phenotyping (1–9 McNeal score) at the seedling stage with 14 diverse *P. hordei* pathotypes. The Farm CPU approach proved effective in mapping both known and several novel BLR QTL and the results were validated using the BayesR analysis. Three distinct EEB lines carrying highly effective distinct ASR were used to develop biparental population to map each of the resistance genes to chromosomal location, the single gene in Russian landrace viz. Caspian (*RphCas*) was shown to be distinct from *Rph21* on the long arm of chromosome 4 H based on allelism tests at the F_3_ stage however close physical proximity to *Rph21* in Ricardo required further sequence haplotype analysis to resolve their independent status.

## Materials and methods

### Plant materials

To identify BLR resistance at the seedling stage, 318 diverse barley accessions were initially selected based on their predicted Eastern Europe origin using available passport data, with seed provided by the Australian Grains Genebank (AGG, Horsham, VIC, Australia). Each of the EEB accessions sourced from the AGG was purified by single-seed descent, with seed harvested from a single plant for phenotypic evaluation and gDNA extraction/genotypic analysis as described in Arifuzzaman et al. (2023).

### Pathogen isolates

Phenotypic assessment at the seedling stage in response to BLR leaf rust was conducted using 12 Australian *P. hordei* pathotypes with diverse virulence phenotypes (Table 1) and two US-derived pathotypes at the primary leaf stage under temperature-controlled conditions. The designation of pathotypes for *P. hordei* in Australia is based on virulence or avirulence of an isolate on a set of standard differential genotypes using the octal notation system (Gilmour 1973). The suffix ‘P+’ or ‘P-’ indicates virulence or avirulence, respectively, on *Rph19*, conferred by the cultivar ‘Prior’ (Park 2003).

**Table 1:** Australian *Puccinia hordei* pathotypes used in this study and their virulence information

Pathotype*	Culture #	Virulent	Year identified
200 P-	512	*Rph8*	Unknown
220 P+ (+ *Rph13*)	577	*Rph5*,* 8*,* 13*,* 19*	2003
242 P+	531	*Rph2*,* 4*,* 8*,* 19*	1998
243 P+	507	*Rph1*,* 2*,* 4*,* 8*,* 19*,* 21*	1992
253 P-	490	*Rph1*,* 2*,* 4*,* 6*,* 8*	1976
4610 P+	491	*Rph4*,* 8*,* 9.i*,* 9.z*,* 19*	1990
5453 P-	560	*Rph1*,* 2*,* 4*,* 6*,* 8*,* 9.i*,* 9.z*,* 10*	2001
5457 P-	626	*Rph1*,* 2*,* 3*,* 4*,* 6*,* 8*,* 9.i*,* 9.z*,* 10*	2013
5457 P+	612	*Rph1*,* 2*,* 3*,* 4*,* 6*,* 8*,* 9.i*,* 9.z*,* 10*,* 19*	2009
5653 P+	542	*Rph1*,* 2*,* 4*,* 6*,* 8*,* 9.i*,* 9.z*,* 13*,* 19*	1999
5477 P-	672	*Rph1*,* 2*,* 3*,* 4*,* 5*,* 6*,* 8*,* 9.i*,* 9.z*,* 10*	2017
5672 P+	639	*Rph2*,* 4*,* 5*,* 6*,* 8*,* 9.i*,* 9.z*,* 13*,* 19*	2015

* Isolate virulence profile of US-derived *P. hordei* pathotypes can be found in Arifuzzaman et al. (2023)

### Leaf rust infection procedure for greenhouse and field experiments

Inoculations using different *P. hordei* pathotypes were conducted in an inoculation chamber located in a separate room from the microclimates used to grow the plants to avoid cross-contamination. The inoculation chamber was washed with tap water for four minutes after every inoculation and all tools were washed in 70% ethanol and rinsed with tap water to avoid contamination across consecutive inoculations. Approximately 10–12 days old seedlings were atomised with inoculum suspension (2 mg of urediniospores/1 mL of Isopar L mineral oil using a pressure pack spray can. Inoculated seedlings were incubated at room temperature for 24 h in a dark room in which mist was generated with an ultrasonic humidifier. After incubation, seedlings were moved to microclimate rooms at 21–23 °C with automated irrigation and natural light.

### Phenotypic assessment of Eastern European Barley accessions

Greenhouse seedlings were scored for response to the *P. hordei* pathotypes at 9–10 days after inoculation. Both the McNeal (1–9) and modified Stakman (0–4) infection-type (IT) scales described by Park and Karakousis (2002) were used for the assessments. Stakman ITs of ‘0’, ‘;’, ‘1’ and ‘2’ on the host indicated resistant responses and ITs of ‘3’ or higher were considered to indicate susceptibility. Disease response and severity of infection were further described using ‘-’ (less than average for the class), ‘+’ (more than average for the class), ‘c’ (chlorosis) and ‘n’ (necrosis). The McNeal 1–9 scores for each of the lines was based on the homogenous ratings of at least 6–8 biological replicates per line and was used for GWAS. In rare instances where heterogeneous ratings were observed within EEB lines these were denoted as missing data for the purpose of GWAS.

### Genotyping of plant materials

Sequence data generated from a targeted genotyping-by-sequencing assay reported previously by Arifuzzaman et al. (2023) was aligned to the Morex v3 genome assembly (Mascher et al. 2021) using a custom NextFlow pipeline ‘shortbread2’ (https://github.com/plantinformatics/shortbread2) for variant discovery, utilising bwa-mem2-v2.2.1 for sequence alignment, fastp-v0.23.4 for adapter and quality trimming, and GATK (gatk4-v4.5.0.0) for calling SNPs. Genotyping was also undertaken from the same genomic DNA source using the Infinium™ Wheat Barley 40 K v1.0 BeadChip (Keeble-Gagnère et al. 2021), with SNP calls anchored to the Morex v3 genome assembly (Jayakodi et al. 2020). SNP-array genotype calls were converted to VCF format and merged with the tGBS variant calls, markers passing filters (biallelic SNP, MAF > 1%, call rate > 80% and < 20% heterozygosity, unique genome position) were retained, resulting in 36,098 SNPs. Missing data was imputed and phased using a NextFlow pipeline utilising Beagle 4.1 and Eagle 2.4.1.

### Genotypic diversity analysis

We previously performed GWAS using the same 318 EEB accessions with the targeted GBS restriction enzyme-based complexity reduction platform (tGBS) (Arifuzzaman et al. 2023). In the present study, the same panel of 318 lines was also genotyped using the Infinium™ Wheat Barley 40 K BeadChip. To assess genetic diversity of the EEB collection relative to accessions stored in the AGG, genotype data for the EEB samples derived from the Infinium™ Wheat Barley 40 K v1.0 BeadChip was combined with raw genotype data for 13,989 diverse accessions in the AGG (Australian Grains Genebank Strategic Partnership 2024), derived from the Infinium™ Wheat Barley 40 K v1.1 BeadChip. This resulted in data for 11,513 SNP markers common in both assays. Principal Component Analysis (PCA) using SNPRelate in R (Zheng et al. 2012; Wickham 2016) was performed on 6,256 markers with > 0.05% MAF and > 95% call rate and SNPs were LD pruned using an r^2^ threshold of 0.7.

### Genome-wide association analysis

GWAS was performed separately for each seedling leaf rust response trait using McNeal 1–9 scores as quantitative phenotypes. The Genome Association and Prediction Integrated Tool (GAPIT version 3.0, R package; http://zzlab.net/GAPIT; Wang and Zhang (2021) was used to analyse the rust resistance data. The GWAS analysis was performed using the FarmCPU (Fixed and Random Model Circulating Probability Unification) method (Liu et al. 2016). FarmCPU increases statistical power and reduces false positives by iteratively applying fixed-effect models to test individual markers and random-effect models to select associated markers. By separating marker testing from kinship estimation, FarmCPU avoids confounding between testing markers and population structure, leading to more accurate detection of true associations.

**Figure:**
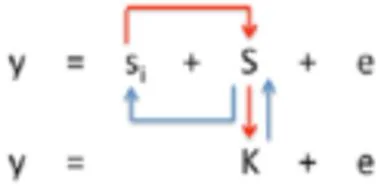

In the equation, y represents the phenotype, s_i_ is the i-th marker (tested one at a time; i = 1…m), S is the set of markers included in the fixed-effect model, K is the kinship matrix derived from associated markers, and e denotes the residual error. SNPs showing minor allele frequencies below 0.01 were eliminated from the dataset. A total of 36,098 polymorphic markers from 318 genotyped accessions were used for GWAS. FarmCPU was fitted using a kinship matrix and the first three principal components as covariates to account for relatedness and population structure, respectively. The Bonferroni correction threshold value of −log10(p) > 5.8 was applied as the cutoff.

### BayesR analysis

The Bayesian modelling (BayesR) definition explicitly allows for some SNPs to have larger estimated variances than methods such as GBLUP, which tends to shrink the variance of causal markers due to the assumption of a common variance (Kemper et al. 2018). As such, BayesR has the potential for more closely approximating the true variance of QTLs. The BayesR model was implemented using BayesR3 software (Breen et al. 2022) with the prior mixture proportions of 94%, 4.9%, 1% and 0.1 of SNPs assigned to effect variances classes of 0, 0.0001, 0.001 and 0.01 the additive genetic variance, respectively. The posterior frequency with which SNPs were assigned to each class can be interpreted as an inclusion probability. The BayesR3 was run for 50,000 iterations, with 25,000 as burn-in. The estimated SNP effects were plotted in R package, ggplot2 (Wickham 2016).

### Genetic studies of inheritance of seedling resistance to leaf rust

Three broadly resistant EEB accessions with distinct infection types were crossed with the susceptible cultivar Gus to study the inheritance of seedling resistance in each progeny. The F_3_ generation populations (Caspian/Gus = 117 F_3_ lines originating from one F_1_ plant; Anska/Gus = 100 F_3_ lines originating from one F_1_ plant; Athenais/Gus = 137 F_3_ lines originating from one F_1_ plant) were assessed at the seedling stage in the greenhouse as described above with the avirulent *P. hordei* pathotype 5457 P+. Following phenotypic assessment, F_3_ segregating families from all genetic crosses were classified based on their response relative to parental controls as either homozygous resistant (HR), segregating (Seg) or homozygous susceptible (HS). To determine the inheritance of resistance all populations were subjected to Chi-squared analysis ($\:{X}^{2}$) to determine the goodness-of-fit of observed segregation with theoretical expected genetic ratios for either monogenic or digenic inheritance. For the purpose of Chi-squared analysis ($\:{X}^{2}$) scores greater or equal to IT = 3 was classified as susceptible.

### RNA extraction and BSR-Seq bioinformatics

To map the seedling resistance locus carried by Caspian, bulked segregant RNA sequencing (BSR-Seq) was performed using the Caspian × Gus F_2_ population (*n* = 160 plants) phenotyped with *P. hordei* pathotype 5457P+. Following phenotypic assessment, plants showing the most clearly resistant and susceptible infection types were selected from the tails of the F_2_ population. Equal quantities of leaf tissue were collected from 38 resistant and 42 susceptible F_2_ individuals and pooled separately to generate resistant and susceptible bulks. Leaf tissue was also collected from the resistant parent Caspian and the susceptible parent Gus for parental RNA-Seq analysis. RNA was extracted from both parents and resistant and susceptible bulks were sequenced using short read Illumina technology at 50 x coverage as described by Chen et al. (2023). BSR-Seq was also performed using the Bowman+*Rph5.e*/Golden Promise F_2_ population as a positive control based on the previously mapped *Rph5.e* locus on chromosome 3HS (Park et al. 2015).

Raw sequencing reads were first assessed for quality using FastQC. Adapter sequences and low-quality bases were removed using Trimmomatic, and filtered reads were aligned to the barley Morex v3 reference genome. SNPs were called jointly across the two parents and the resistant and susceptible bulks using GATK. Variant calls were filtered to retain high-confidence biallelic SNPs with sufficient read depth in both bulks and clear polymorphism between the parental lines. Allele frequencies were calculated for each informative SNP in the resistant and susceptible bulks. The SNP frequency ratio, between the resistant and susceptible bulks, was then calculated across the genome to identify regions enriched for the Caspian allele in the resistant bulk and depleted in the susceptible bulk. SNP frequency values were plotted according to their physical positions on the Morex v3 reference genome, and chromosome-level patterns were inspected to identify genomic regions showing strong divergence between bulks.

### Conventional BSA and 15 K SNP genotyping analysis

To validate the chromosome 4HL location of *RphCas* identified by BSR-Seq and to further refine the candidate region, a conventional BSA was performed using DNA bulks derived from phenotypically validated F_3_ families. The F_2_ plants used to generate the resistant and susceptible RNA bulks were advanced to the F_3_ generation and progeny tested with *P. hordei* pathotype 5457P + to confirm their genetic classification. Seven homozygous resistant and six homozygous susceptible bulks (equal tissue from three plants each from three different HR/HS families per bulk) derived from F_3_ families were constructed. The parental lines and DNA bulks were genotyped using a barley 15 K SNP genotyping platform at the Julius Kühn-Institut/associated genotyping facility in Germany. Genotype calling was performed using the Illumina Infinium GenomeStudio workflow. Genotype calls were exported as nucleotide calls together with marker chromosome and physical position information based on the Morex v3 reference genome. SNPs polymorphic between Caspian and Gus were retained as informative markers for BSA. For each informative marker, the parental allele pattern was compared across the homozygous resistant and homozygous susceptible bulks. Fully fixed markers were defined as those for which all resistant bulks carried the Caspian allele and all susceptible bulks carried the Gus allele. Nearby parent-polymorphic markers showing the same pattern but containing occasional mixed or failed calls were considered supportive.

## Results

### Population structure of the Eastern European barley panel

**Fig. 1:**
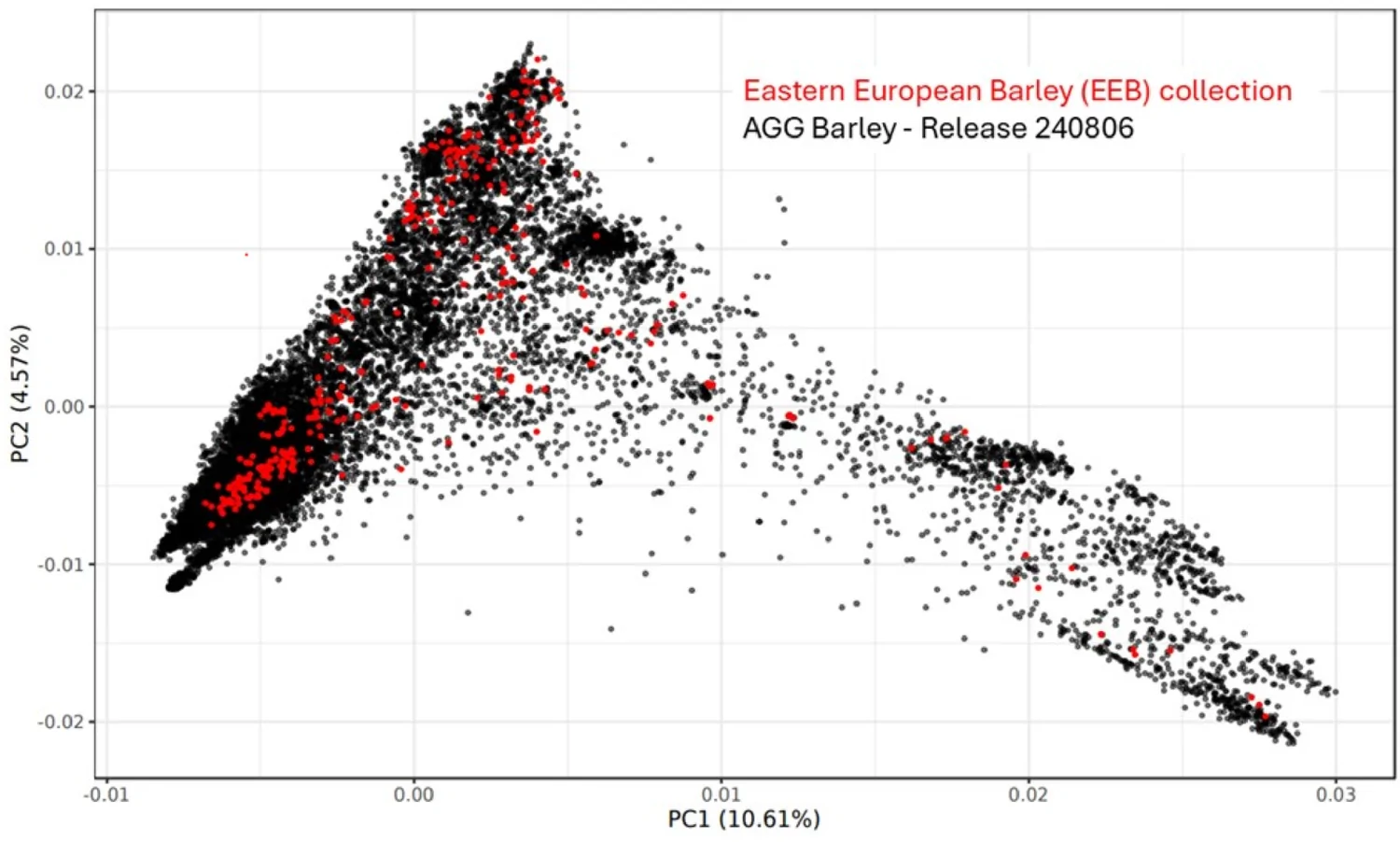
Principal component analysis (PCA) of globally diverse barley genotypes and selected Eastern European barley (EEB) lines (red) in the Australian Grains Genebank

The principal component analysis (PCA) was used to examine the genetic structure of a subset of barley Plant Genetic Resources from the AGG (Australian Grains Genebank 2024), including globally diverse accessions (black) and selected EEB lines (red). The first two principal components together accounted for 15.36% of the total genetic variation and clearly separated the accessions (Fig. 1).

The EEB subset was initially expected to represent a relatively narrow but distinct pool of diversity. However, when analysed alongside the broader AGG collection, the EEB accessions occupied a wider portion of the global diversity landscape than anticipated. Most EEB accessions clustered on the left-hand side of the PCA plot, consistent with the position of other European material. Notably, several accessions grouped on the opposite side of PC1, within the region typically associated with Asian barley diversity. These accessions were recorded in their passport data as originating from Georgia, Armenia, and the former USSR, which is consistent with their genetic separation from the main European cluster (Fig. 1).

### Phenotypic leaf rust response under controlled greenhouse conditions

**Fig. 2:**
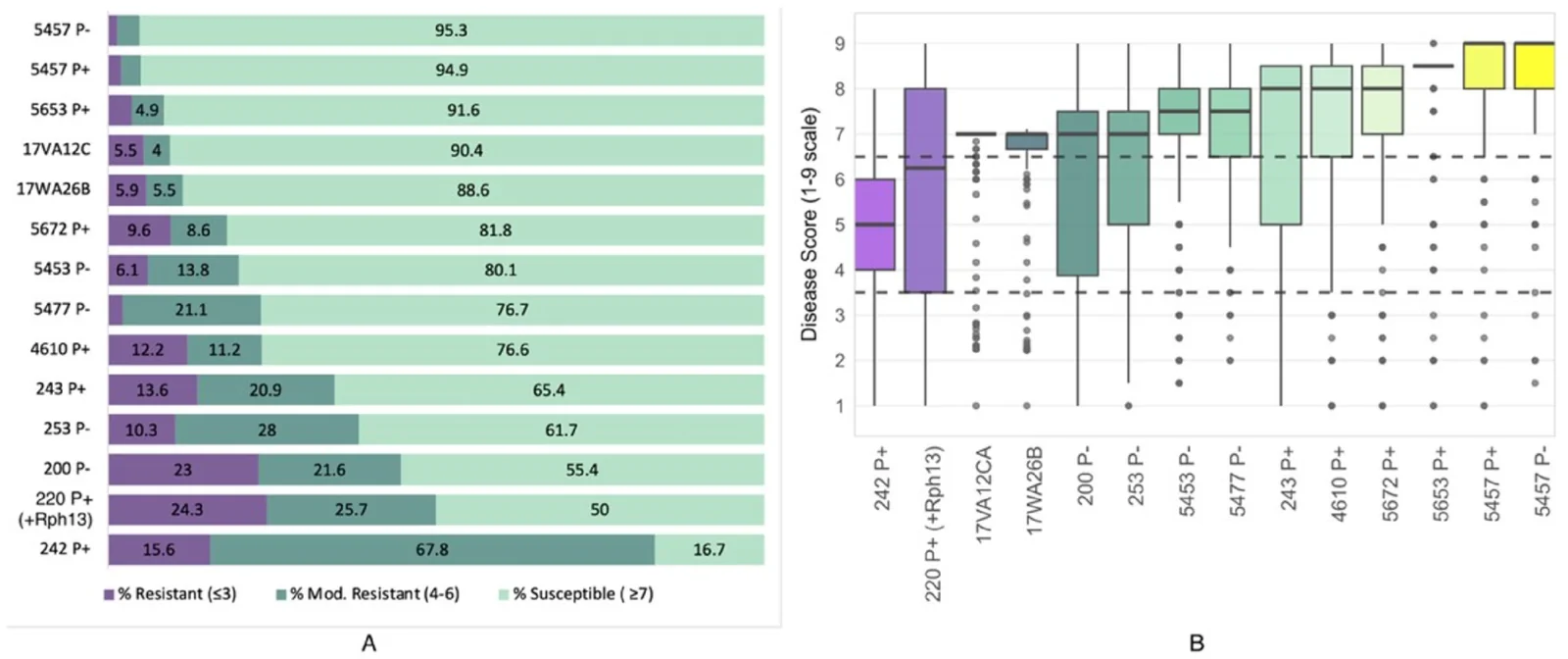
(**A**) Frequency distribution and (**B**) Boxplot of phenotypic response of EEBs accessions against 14 *Puccinia hordei* pathotypes used in this study. Data based on quantitative 1-9 McNeal scale data recorded 10 days post infection

The virulence and avirulence profiles of the 12 Australian (Table 1) and two US derived *P. hordei* pathotypes used for seedling phenotyping of the EEBs were selected to capture virulence to a broad range of known *Rph* genes and to enable the detection of both known and potentially novel resistance alleles in the EEB panel. The phenotypic distribution frequencies for all leaf rust traits are shown in Fig. 2. Most traits were skewed towards susceptibility, indicating that only a limited number of rust resistant accessions that were broadly resistant to most pathotypes (Fig. 2A). The phenotypic response results revealed that increased susceptibility in the EEBs to the *P. hordei* pathotypes tested was correlated with increased virulence of the *P. hordei* pathotypes. For example, the most virulent pathotypes based on the response across the EEB accessions were 5457P+, 5457P- and 5653P+, with a mean McNeal score > 8, whereas in contrast pathotypes 200P-, 220P+ (+ *Rph13*) and 242P + had a mean McNeal scores of < 5 (Fig. 2B). This pattern is largely explained by the presence of known *Rph* resistance alleles such as *Rph1*,* Rph2*,* Rph3*,* Rph4*,* Rph9.am* and *Rph12* which are common among global barley accessions either singly or in combination. Overall, the mean McNeal score indicated that very few lines (1.3%) were resistant (scores 1–3), whereas 21% were moderately resistant (scores 4–6) and 77% were susceptible. Three accessions of Greek origin (Athenais, Rodon and PI 249934) were broadly resistant (score ≤ 3) to all pathotypes, except for 242P+ (score 7), indicating the presence of the same uncharacterised resistance allele and pathotype specificity. Furthermore, two lines from Russia were broadly resistant to all pathotypes tested but expressed distinct infection types suggesting that they carried one or more distinct BLR resistance alleles (Figure S1). Anska was completely immune (IT 0;) to all 14 pathotypes tested with the lowest mean McNeal score (1.4) of all EEB accessions, whereas Caspian produced an intermediate IT (;1-C, McNeal 3.7), suggesting that it represents a unique, uncharacterised BLR resistance allele among the EEBs. Numerous EEBs had mean McNeal scores between 3 and 5, however, this was largely explained by the likely presence of *Rph3* in these accessions and its low infection type and *Rph3* virulence in only three of the 14 *P. hordei* pathotypes used in this study.

### Marker trait associations

**Fig. 3:**
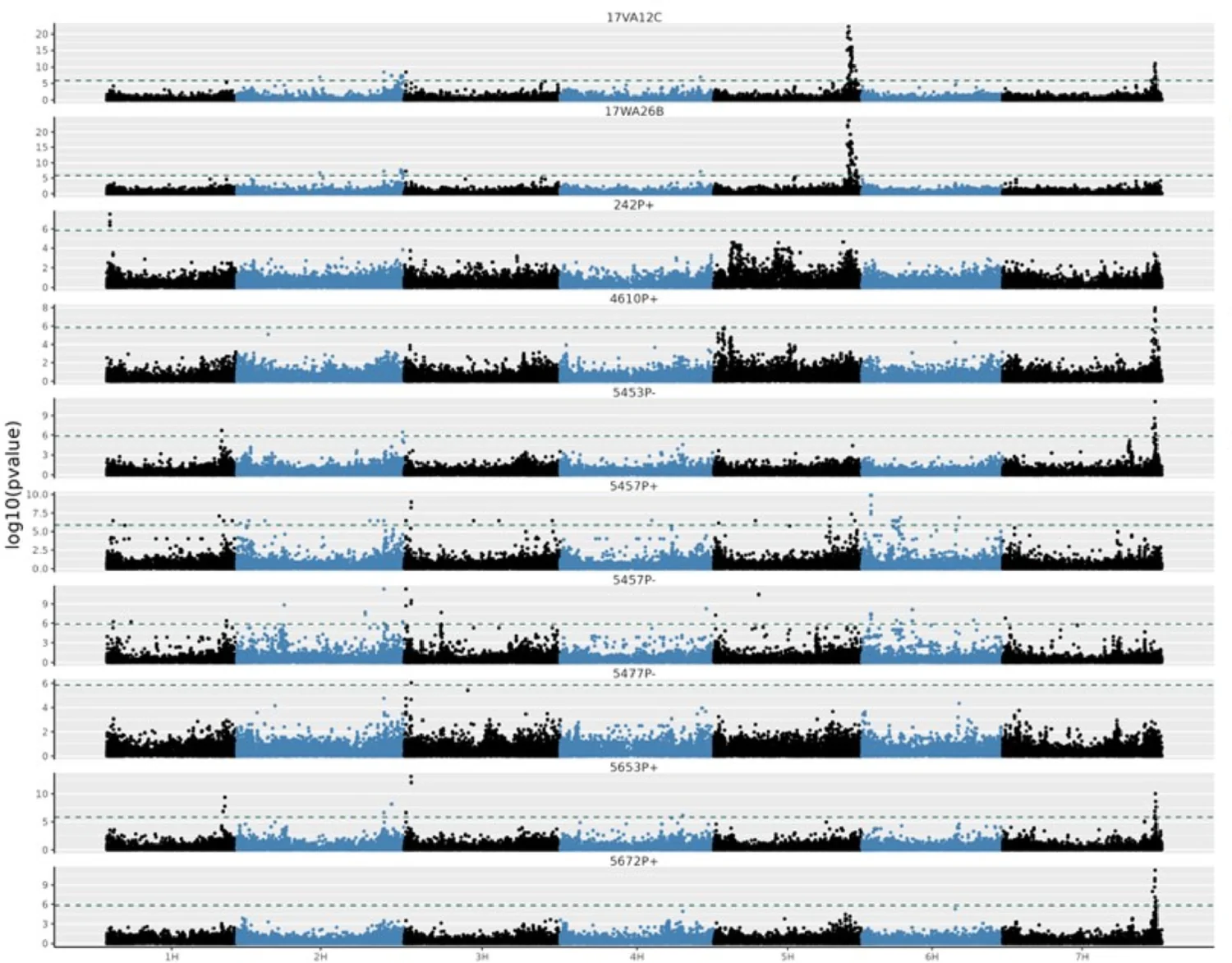
Manhatten plots of genome-wide association mapping of barley leaf rust resistance in the 318 EEB accessions at the seedling in response to 10 pathogenically diverse *Puccinia hordei* isolates using the FarmCPU model in GAPIT. The green horizontal line indicates the significance threshold (*p* < 0.001 [- log(10)(p) = 5.8] according to the Bonnoferri method)

GWAS revealed heterogeneous association patterns across the tested pathotypes, with signal profiles broadly reflecting their virulence and avirulence spectra. This indicates that the analysis detected marker–trait associations corresponding to known genetic determinants of leaf rust resistance, as well as additional genomic regions potentially associated with novel resistance loci (Table 2). Manhattan plots for all analysed pathotypes are shown in Fig. 3 and Figure S2 (circular Manhatten). Among the 14 seedling resistance traits, clear association signals were observed for 10 pathotypes, with one or more genomic regions exceeding the Bonferroni significance threshold − log₁₀*p* > 5.8. Only associations above this threshold were considered robust. A cluster of SNPs was detected for the 17VA12C and 17WA26B traits in a genomic region that co-localizes with the previously reported *Rph12* locus on the long arm of chromosome 5H as previously reported (Arifuzzaman et al. 2023). Two additional peaks were identified on chromosome 5HS, one associated only with 4610P+ and another associated with both 5457P − and 5457P+, most likely corresponding to known catalogued genes *Rph2* and *Rph20*, respectively.

In contrast, a weak but significant peak on chromosome 7HL in response to 5453P- is most likely *Rph19* based on it being one of the only BLR pathotype traits with significant peaks that is avirulent to *Rph19* previously mapped to 7HL. Significant peaks on the top of chromosome 3HS correspond to the position of both *Rph5* and *Rph7*, however the peaks do not correspond completely to the virulence/avirulence spectra of the isolates and none of the EEB lines had the distinctive *Rph5* or *Rph7* infection type. The analyses also detected significant novel marker–trait associations underlying resistance to several highly virulent isolates on chromosomes 1HL (*RphQTL1*), 2HL (*RphQTL2*) and 3HS that likely represent novel broadly effective resistance loci (Table 2).

**Table 2:**
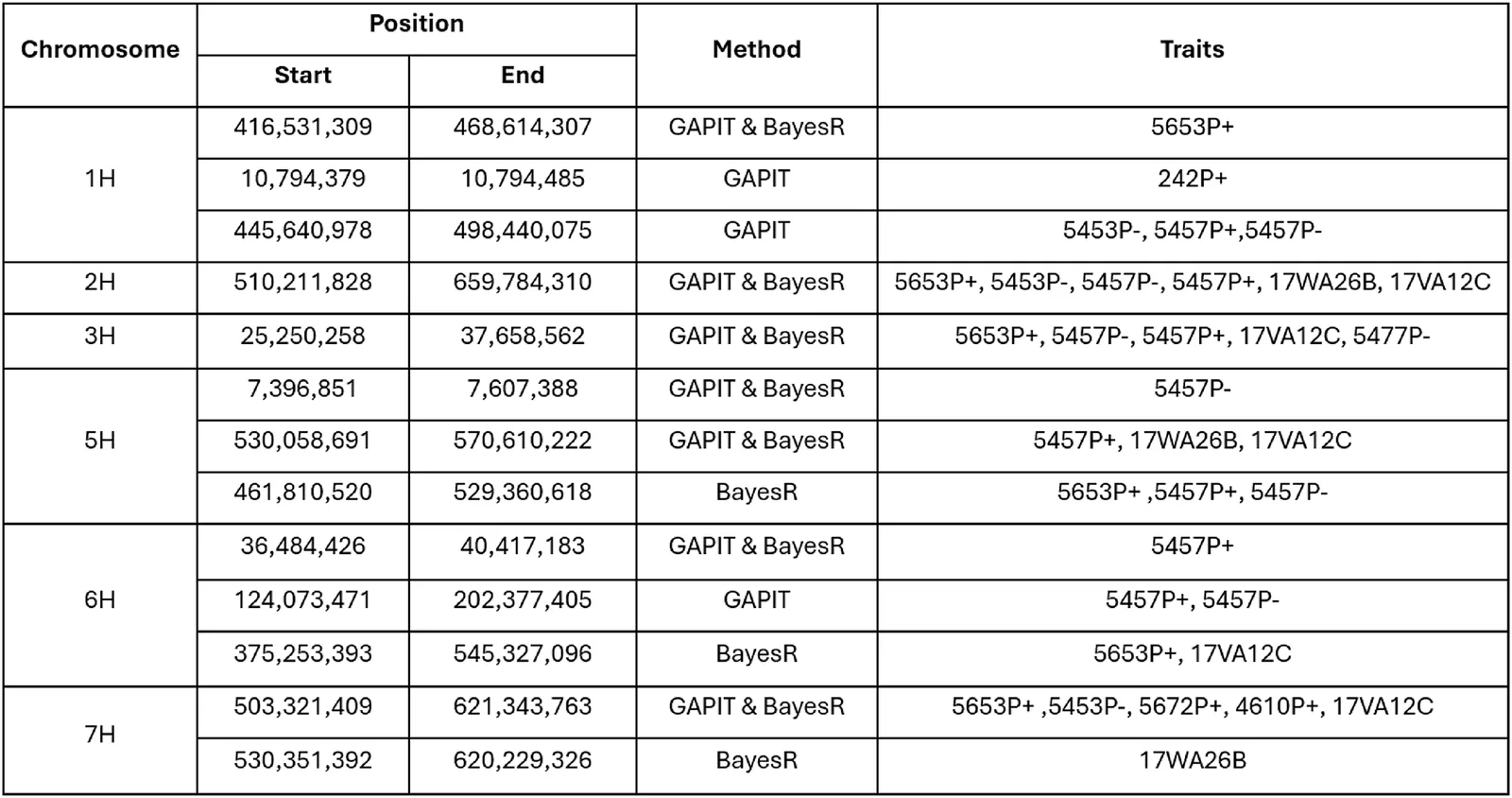
Significant chromosomal regions associated with BLR resistance detected by different methods against investigated *Puccinia hordei *pathotypes

### SNP effects and BayesR analysis

Implementing BayesR3, several of the major associations between SNPs and BLR pathotype resistance traits identified in the GWAS study were detected. The correspondence between methods, provides additional support for the SNP–trait relationships detected in the initial GAPIT GWAS analysis and increase confidence in the identified genomic regions (Figure S3). The results consistently identified SNPs on the long arm of chromosome 7H, showing higher effects in response to a group of pathotypes (4610P+, 5453P-, 5672P+, 5653P+, 242P+, and 17VA12C), were included in the model with higher probability. This pattern was also previously demonstrated using the GWAS method and based on the detection of the cloned *Rph3* gene.

Additional SNPs with relatively higher effects were also detected on the long arm of chromosome 7H in response to pathotype 5453P−. Although these SNPs were located close to the broader 7HL-resistance associated region, their slightly different position suggests the presence of a neighbouring but potentially distinct signal, most likely to be *Rph19* based on pathotype specificity of 5453P-. On the short arm of the chromosome 5H, SNPs with higher effects were identified against 4610P + in the location of *Rph2.* The results revealed SNPs putatively contributing to resistance against pathotypes 5457P+, 17WA26B and 17VA12C located on long arm of chromosome 5H. A similar group of SNPs with elevated effect was also detected using GWAS, supporting concordance between the two methods. Both GWAS and BayesR also revealed SNPs on short arm of the chromosome 5H against 5457P-. The method further detected the significant SNP association on the long arm of the chromosome 1H, revealed by GWAS method for 5653P+. Additionally, the BayesR method revealed several unique regions that entered the model with higher probability. For example, on the short arm of chromosome 4H, a SNP with a relatively strong effect was identified as contributing to resistance to isolate 5672P+. The analyses also detected marker–trait associations underlying resistance to isolate 5653P+ on the long arm of chromosome 6H as well as on the short arm of chromosome 2H. Furthermore, on the short arm of chromosome 6H, SNPs with higher effects were identified in response to isolate 5457P+. Taken together, these findings demonstrate both strong agreement with the GWAS results and the added capacity of the BayesR approach to detect additional, potentially novel genomic regions associated with resistance.

### Inheritance of BLR resistance in segregating biparental populations

The five accessions with broadly effective distinct seedling resistance (McNeal score < 3.5) identified through multi-pathotype testing were likely not able to be detected through the GWAS analysis therefore R/S F_3_ populations were produced and phenotyped using the 5457P+ pathotype. In the first instance, all three F_3_ populations were rust tested with *P. hordei* pathotype 5457P + and Chi-squared analysis was used to determine the best fit to either a monogenic (1:2:1) or digenic (7:8:1) inheritance model. BLR resistance in Anska was inconclusive likely due to segregation distortion, Athenais/Gus fitted a digenic model whereas *RphCas* was inherited as a single dominant gene (Table 3). Both Athenias/Gus and Anska/Gus F_3_ populations were also rust tested with the *Rph5* virulent *P. hordei* pathotype 220 P+ (+ *Rph13*), Athenias/Gus segregated for two genes but Anska/Gus neither fitted a one or two gene segregation and therefore the mode of inheritance was inconclusive in Anska (Table 3).

**Table 3:** Genetic analysis based on biparental populations developed to determine the inheritance of barley leaf rust resistance

Genotype	*P*. hordei pathotype	HR count	SEG count	HS count	Ratio	Chi-squared	*P*-value	Interpretation
Athenais/ Gus F_3_	5457P^+^	59	85	12	1:2:1 7:8:1	29.577 2.401	< 0.01** > 0.30	No fit Fit
	220P^+^ +*Rph13*	56	60	12	1:2:1 7:8:1	30.75 2.25	< 0.01** > 0.32	No fit Fit
	242P^+^	0	0	120	1:2:1 7:8:1	360 1800	< 0.01** < 0.01**	No fit No fit
Caspian/Gus F_3_	5457P^+^	35	52	30	1:2:1 7:8:1	1.872 76.231	> 0.39 < 0.01**	Fit No fit
Anska/Gus F_3_	5457P^+^	28	53	8	1:2:1 7:8:1	12.236 5.764	< 0.01** > 0.05	No fit Inconclusive
Anska /Gus F_3_	220P + + *Rph13*	35	49	16	1:2:1 7:8:1	7.26 16.8	0.02* < 0.01**	No fit No fit
Caspian/Ricardo F_3_	5457P^+^	41	59	10	7:8:1	2.766	> 0.25	Fit

*Significant at *p* < 0.05 (χ²critical = 5.991, df = 2); **significant at *p* < 0.01 (χ²critical = 9.21, df = 2)

### Mapping BLR seedling resistance in Caspian using bulk segregant analysis

**Fig. 4:**
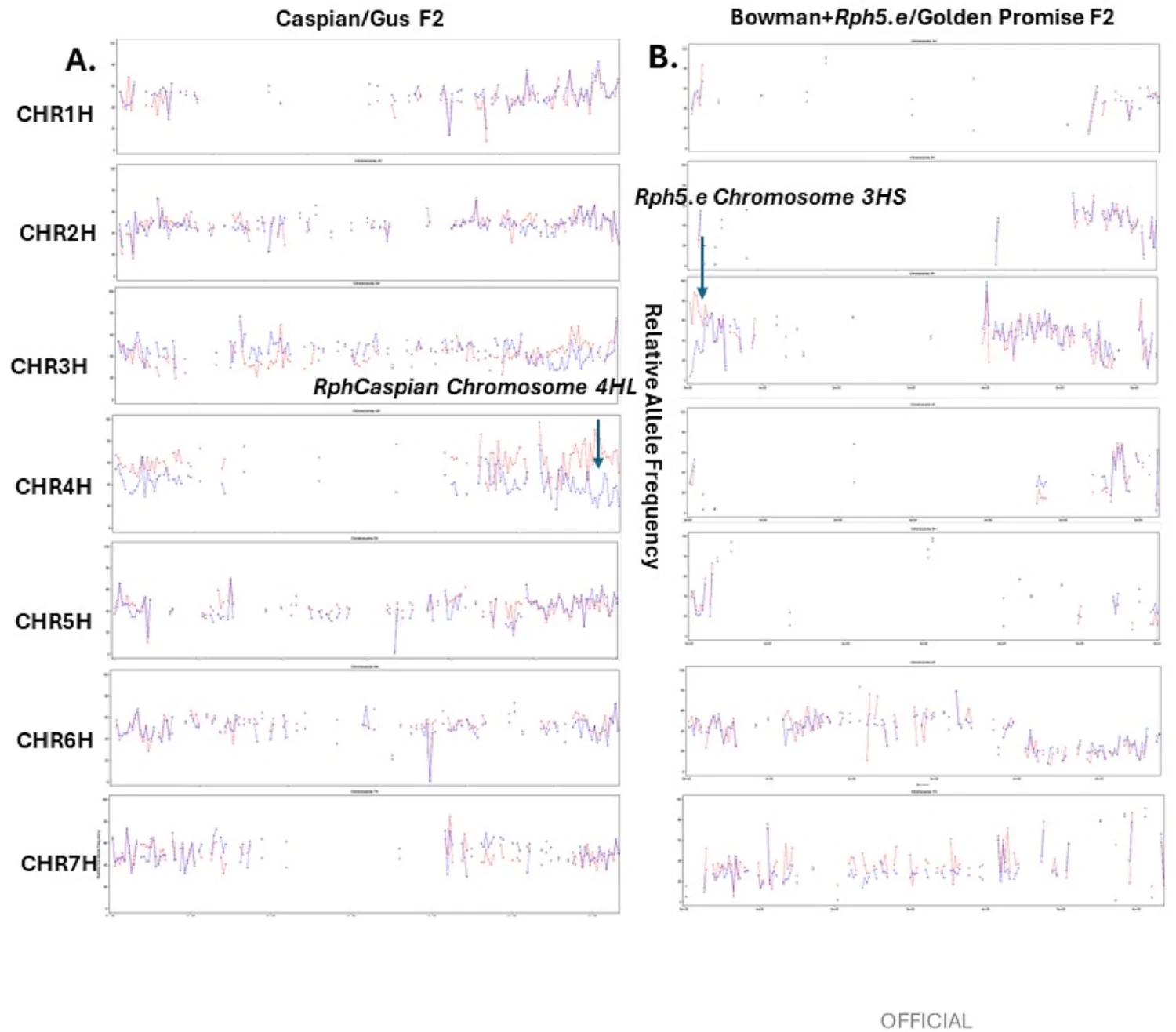
Bulk Segregant RNA-Seq analysis visualising only SNPs with 100x coverage in both pools. Pooled RNA bulks of R and S and each parent were sequenced at 50x coverage mapping *RphCaspian* to Chromosome 4HL in the Caspian/Gus F_2_ population and *Rph5.e* to its previously confirmed map location of 3HS in the Bowman+*Rph5.e*/Golden Promise F_2_ population (Park et al. 2015). *Rph5.e* was used as a control for the BSR-Seq analytical approach. The map locations of *RphCaspian* (4HL) and the control *Rph5.e* (3HS) are denoted by arrows where the difference in relative allele frequency is the greatest between resistant and susceptible bulks

**Fig. 5:**
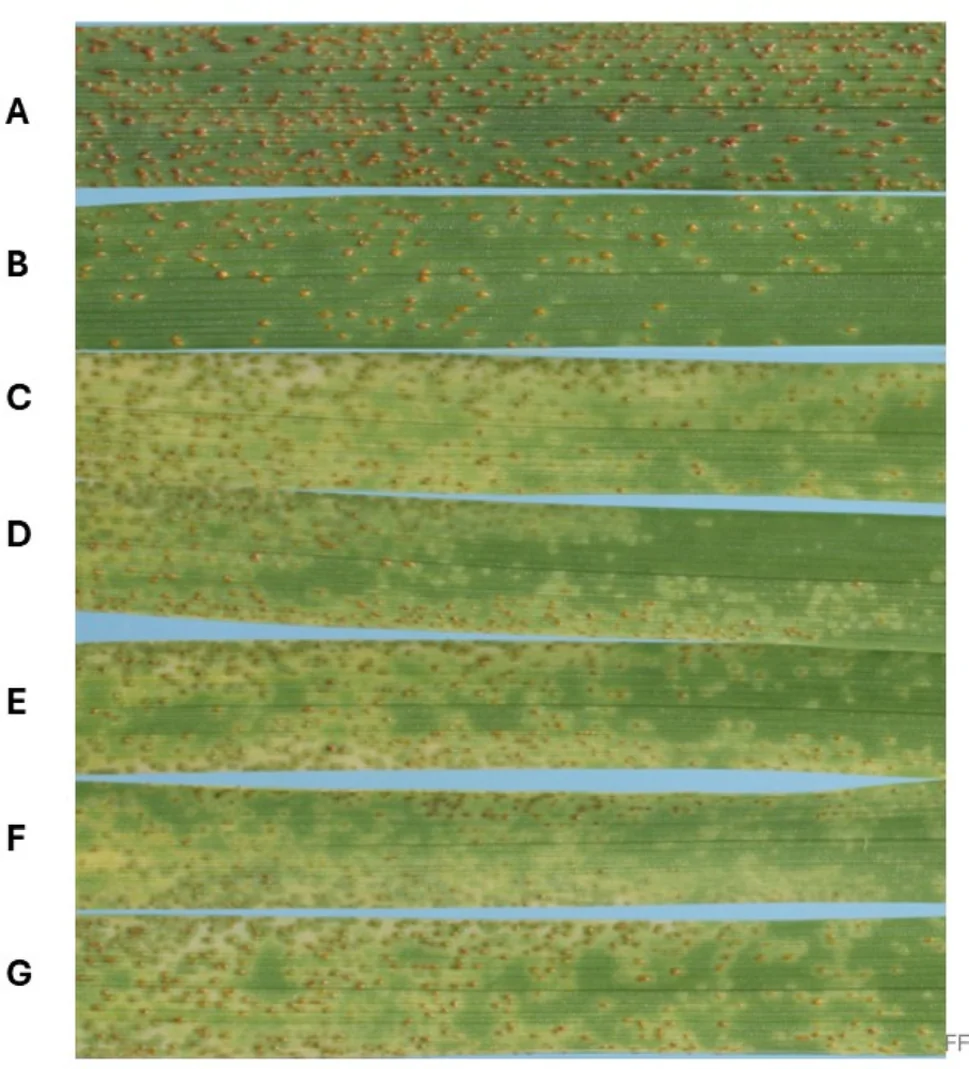
Phenotypic assessment in selected lines of the Caspian (*RphCaspian*) x Ricardo (*Rph21*) F_3_ population as part of a test of allelism between two genes mapping to the long arm of chromosome 4H including *Rph21* Sandhu et al. 2012 and *RphCaspian* in the present study. Phenotypic assessment of HR (C, D and E) and HS line (B), resistant parents F (Caspian), G (Ricardo) and universal susceptible line Gus (A) was performed 10 days post inoculation using a *Puccinia hordei* pathotype 5457P+

RNA-Seq was performed on the parents and bulks followed by SNP frequency ratio analysis were plotted using the Morex V3 reference to identify a disparity in SNP frequency (Fig. 4). BSR-Seq analysis mapped *RphCas* to the long arm of chromosome 4H in a similar region to the previously characterised *Rph* allele *Rph21* from Ricardo. Using the same BSR-Seq strategy we re-mapped the *Rph5.e* BLR resistance allele segregating in the Bowman+*Rph5.e*/Golden Promise F_2_ population to the short arm of chromosome 3H as a positive control (Fig. 4). The results of the BSR-Seq analysis were further validated by progeny testing the same F_2_ plants used for the R and S bulks at the F_3_ stage with 5457P+. Based on the success of the progeny testing classical BSA was performed by genotyping resistant and susceptible bulks and the parents using 15K SNP array platform. The BSA analysis delineated the chromosome location of *RphCas* to a 6.59 Mb region on 4HL supporting the BSR-Seq results based on the physical location of the two flanking markers (Table S1). A test of allelism was performed by crossing Ricardo with Caspian followed by rust testing of F_3_ families with *P. hordei* pathotype 5457P+ that is avirulent to both *RphCas* and *Rph21* (Fig. 5). The segregation pattern, presence of 10 susceptible F_3_ lines and Chi-Squared analysis suggests that *RphCas* is a distinct newly identified resistance gene on Chromosome 4HL distinct from *Rph21* based on segregation closely conforming to a digenic inheritance model (7 HR: 8 Seg: 1 HS, Table 3). The race-specific *Rph27* gene was mapped to the short arm of chromosome 4H, in contrast both *RphCas* and *Rph21* map to the long arm of chromosome 4H in a broadly similar physical window. *Rph21* was originally mapped using BSA using a small set of polymorphic markers therefore the resolution of its physical location is low however as the physical interval is overlapping further haplotype and sequence based analysis will be performed to determine whether these loci are indeed truly distinct.

## Discussion

The introduction of genetic diversity into breeding programs is shaped by scientific exchange, germplasm availability and historical patterns of crop movement. Genebanks therefore represent essential reservoirs of allelic diversity that can be used to identify novel sources of resistance to fungal diseases and other important agronomic traits (König et al. 2012; Arifuzzaman et al. 2023; Yuan et al. 2024). In Australia, the Australian Grains GeneBank (AGG) is particularly valuable because strict biosecurity requirements can limit the direct introduction of exotic germplasm. Mining existing genebank collections therefore provides a practical route to broaden the resistance base available for barley improvement.

We recently reported on the utility and identification of novel resistance alleles to exotic rust pathogens in a collection of diverse barleys selected based on their proposed eastern European (EEBs) origin (sourced from the AGG) using targeted GBS and using a MLM GWAS approach (Arifuzzaman et al. 2023). The EEB collection was preferentially selected based on Eastern European origin using passport data available at the AGG, however the GPS location of collection was not accessible for all lines and typically place of origin can be misleading or accessions are commonly renamed. In this study all 318 accessions were re-genotyped using the Infinium™ Wheat Barley 40 K BeadChip, permitting direct comparison on a PCA plot of recently genotyped subset of 13,989 diverse AGG accessions. The new array is designed to maximally capture haplotypic diversity in globally diverse wheat and barley germplasm while minimizing ascertainment bias. Comprising mostly biallelic markers that are designed to be species-specific and single-copy, the array permits highly accurate imputation in diverse germplasm to improve the statistical power of genome-wide association studies (GWAS) and genomic selection. Plotting the EEBs amongst the AGG accessions indicated that although there was distinct expected clustering of EEBs, several EEBs clustered with barleys from genetically diverse geographic origin. This highlights the importance of genebanks genotyping their collections to enable users to place their selected germplasm in context.

Several studies have successfully combined detailed multi-pathotype phenotypic screens (using Stakman 0–4 scale) of diverse barley accessions sourced from the AGG for gene postulation of new effective *Rph* alleles followed by genetic analysis of biparental mapping (Derevnina et al. 2013; Elmansour et al. 2017; Mehnaz et al. 2021b). The present study used the McNeal 1–9 scale to phenotype all 318 accessions that is more amenable to quantitative genomic analysis such as GWAS. This approach permitted the identification and mapping of novel and known race-specific *Rph* genes present in the population (including *Rph2*,* Rph3*,* Rph4*,* Rph12*,* Rph9.am* and *Rph19*) in addition to QTL of both major and minor effect that may likely represent partial resistance that are often unable to be identified using the 0–4 scale. By combining and performing curations from two different genotypic datasets including tGBS (Arifuzzaman et al. 2023) and the more recent Infinium™ Wheat Barley 40 K BeadChip we performed GWAS using FarmCPU and the significant peaks identified, using the strict Bonferroni threshold and the BayesR approach. The cloned *Rph3* locus (Dinh et al. 2022) was prevalent in the EEBs and served as an effective positive control for our GWAS results. The *Rph3* peak on the long arm of chromosome 7H was detected using both FarmCPU and BayesR only in response to *Rph3*-avirulent *P. hordei* pathotypes. Similarly, a significant peak proximal to *Rph3* also on 7HL was identified only with the *Rph19*-avirulent *P. hordei* pathotype and is therefore predicted represent a refined map location for *Rph19* relative to the QTL mapping study (Park and Karakousis 2002).

Other known *Rph* genes such as *Rph4* and *Rph12* were mapped to chromosome 1HS and 5HL, respectively, only in response to avirulent pathotypes. This supports that the quantitative approach used to phenotype the EEBs with 14 diverse pathotypes was effective to determine the genetic architecture of BLR resistance using a population genomic approach. Despite this, no significant GWAS peaks using FarmCPU were identified for four highly avirulent isolates (200P-, 220P+(+ *Rph13*), 253P- and 243P+). However, several markers were found to be in close LD at expected positions mapping to *Rph2*, *Rph3* and *Rph12* but failed to exceed the strict Bonferroni significance threshold. Two genomic regions on the long arm of chromosome 1H and 2H were identified in response to the most virulent pathotypes used in the study that do not correspond to any of the known *Rph* genes in cultivated barley. Previous studies mapping partial resistance to BLR either by measuring visible infection sites per unit area or increased latency period in the Vada/L94 population have identified partial resistance genes on chromosome 2HL (Qi et al. 1998; *Rphq2* and *Rphq11*), however only *Rph26* from *Hordeum bulbosum* has been mapped to the long arm of chromosome 1H. The novel QTL identified in the present study will be further investigated and characterised in future studies.

One limitation of using a GWAS approach is that rare or distinct resistance alleles present in a single or a few related accessions are often not detected due to minimum allele frequency. In the present study three phenotypically distinct yet widely effective resistant sources were identified from Russian (Caspian and Anska) and Greek (Rodon, Athenais and PI 249934) origin. Distinct incompatible infection types were however identified in the Russian landraces Anska and Caspian, therefore, to determine the inheritance and map the distinct *Rph* alleles three different segregating populations were produced. Genetic analysis determined a simple monogenic inheritance in Caspian whereas the resistance in both Anska and Athenais fitted a two-gene inheritance model. Russian landrace Anska was completely immune to all *P. hordei* pathotypes assessed in this study. This response has previously been due to the presence of *Rph5* in combination with either *Rph2* or *Rph3*, resulting from the rare double avirulence in Australian *P. hordei* populations. Further investigation into the marker haplotype of Anska determined it carries both QTLs on 1HS and 3HS that largely explain the low infection type. Based on the close genetic relationship of the three resistant Greek accessions, similarity of the infection types and shared pathotype specificity we hypothesised they contained the same BLR resistance allele. Remarkably all three Greek accessions carried four novel QTLs identified in this study including 1HL, 2HL, 3HS and 6HS (Table S2).

To map *RphCas*, we used a bulk segregant RNA-Seq approach to rapidly assign the locus to a chromosome arm. The BSR-Seq approach has been used extensively in cereal crops especially wheat to map agronomic traits of interest, however this study is the first application of the approach to map BLR to our knowledge. *RphCas* mapped to a 6.58 Mb physical region (576-583 Mb) on the long arm of chromosome 4H. Two *Rph* genes have been mapped to date on chromosome 4H. *Rph27* was mapped by Rothwell et al. (2020) to the telomere on the short arm of 4H using DArT-Seq marker platform and the other more relevant locus is *Rph21* from Ricardo that maps to the long arm of 4H using BSA (Sandhu et al. 2012). Based on the Morex v3 reference genome, *Rph21* is located on 4H between 569.2 and 587.5 Mb, spanning an interval of ~ 18.3 Mb overlapping with 6.58 Mb physical region of *RphCas* (576–583 Mb). To ascertain whether two loci are independent, we performed allelism tests which demonstrated that segregation did not deviate from two gene segregation (7:8:1 *P* > 0.25) and two genes are likely independent. Nevertheless, a loose linkage cannot be excluded based on these data, as the population size may have limited the power to distinguish recombination frequencies close to 50% from complete independent assortment. Future work will focus on fine mapping both loci with the aim of further resolving the haplotypic patterns and physical location of both genes and determining whether they are indeed distinct.

In conclusion, performing GWAS combining the Infinium™ Wheat Barley 40 K BeadChip and tGBS data improves the utility of our dataset as the most significantly associated SNPs closely associated with both novel and previously identified *Rph* alleles can be added to future iterations of the SNP array. The same SNP array has also been used to genotype all barley accessions of the AGG, thus permitting haplotype-based predictions of BLR resistance in any barley accession informing the efficient selection of core collections. In future, characterisation of resistance gene-linked haplotypes in this way can assist with more targeted selection of accessions which do not carry known genes, accelerating the discovery of novel resistance sources. Further studies will involve fine mapping and both sequence and haplotype based analysis of *Rph21* and *RphCas* to determine whether they are distinct BLR resistance alleles on chromosome 4HL.

## Supplementary Information

Below is the link to the electronic supplementary material.

Supplementary Material 1

Below is the link to the electronic supplementary material.

Supplementary Material 2

## Data Availability

The dataset analysed during this study is either publicly available or is available by request through correspondence p.dracatos@latrobe.edu.au.
