# Supplementary material for: Genome-wide characterisation of leaf rust resistance loci in an Eastern European barley collection
Source: Theor Appl Genet. 2026 Oct 17;139(11):307. doi: 10.1007/s00122-026-05405-4 (PMC13676509; doi:10.1007/s00122-026-05405-4)
Supplement: Supplementary file 1 [file 122_2026_5405_MOESM2_ESM.docx]

**Supplementary Figures**
